# Re-examination of Rule-Based Methods in Deidentification of Electronic Health Records: Algorithm Development and Validation

**DOI:** 10.2196/17622

**Published:** 2020-04-30

**Authors:** Zhenyu Zhao, Muyun Yang, Buzhou Tang, Tiejun Zhao

**Affiliations:** 1 Harbin Institute of Technology Harbin China; 2 Harbin Institute of Technology Shenzhen China

**Keywords:** ensemble learning, deidentification, transformation-based error-driven rule learner

## Abstract

**Background:**

Deidentification of clinical records is a critical step before their publication. This is usually treated as a type of sequence labeling task, and ensemble learning is one of the best performing solutions. Under the framework of multi-learner ensemble, the significance of a candidate rule-based learner remains an open issue.

**Objective:**

The aim of this study is to investigate whether a rule-based learner is useful in a hybrid deidentification system and offer suggestions on how to build and integrate a rule-based learner.

**Methods:**

We chose a data-driven rule-learner named transformation-based error-driven learning (TBED) and integrated it into the best performing hybrid system in this task.

**Results:**

On the popular Informatics for Integrating Biology and the Bedside (i2b2) deidentification data set, experiments showed that TBED can offer high performance with its generated rules, and integrating the rule-based model into an ensemble framework, which reached an F1 score of 96.76%, achieved the best performance reported in the community.

**Conclusions:**

We proved the rule-based method offers an effective contribution to the current ensemble learning approach for the deidentification of clinical records. Such a rule system could be automatically learned by TBED, avoiding the high cost and low reliability of manual rule composition. In particular, we boosted the ensemble model with rules to create the best performance of the deidentification of clinical records.

## Introduction

### Background

Electronic health records (EHRs) are rich resources for clinical research in which a large amount of medical knowledge is contained. To protect the privacy of patients, EHRs cannot be directly accessed by researchers without deidentification (ie, removing the information that may reveal the patient’s identity). According to the Health Insurance Portability and Accountability Act (HIPAA) of the United States, 18 categories of protected health information (PHI) must be removed before the release of EHRs, such as name, age, and location, which brings big challenges to the process of deidentification.

Deidentification is conventionally processed manually, with crowd-sourced workers tagging the PHI and removing it. This would be prohibitively expensive in terms of manpower considering the existing large scale of the clinical corpus. With the help of natural language processing technology, automatic deidentification becomes possible. To encourage innovations in this field, in 2006, 2014, and 2016, three deidentification shared tasks were organized by Informatics for Integrating Biology and the Bedside (i2b2). In these shared tasks, most approaches take deidentification as a sequence-labeling problem aimed at generating the proper label to each token in the text [1].

### Task Formulation

Formally, given a sequence *S* = (*s*_1_, *s*_2_, . . ., *s_n_*) of length *n* that needs to be tagged, the target of a tagger is to properly generate a tag *t_i_* for the *i*th token *s_i_* to form a tag sequence *T* = (*t*_1_, *t*_2_, . . . , *t_n_*). As one PHI entity might span multiple tokens, the output sequence *T* follows a format that indicates the inside, outside, and begin (IOB) of a PHI.

For example, given the sentence “*Harlan Oneil is a 43 years old gentleman*”, the outputs of our system should be “*B(NAME) I(NAME) O O B(AGE) O O O*”. The first two tags *B(NAME)* and *I(NAME)* will be merged into a PHI entity, and the fifth tag is a single-token PHI.

### Prior Work

Various methods have been designed for deidentification. Methodologically, current solutions to the deidentification of EHRs can be summarized into three categories: rule-based methods, learning-based methods, and ensemble approaches. Early research in this task was mostly based on rules, such as Sweeney et al [2] and Gupta et al [3]. The rule-based systems used dictionaries and hand-crafted rules derived by medical expertise, which are hard to transfer to other domains. With the rapid growth of machine learning methods, researchers quickly switched to learning-based methods including support vector machine (SVM) [4], decision tree [5], and conditional random field (CRF) [6], and recent deep learning models like recurrent neural network (RNN) [7], long short-term memory (LSTM)-CRF [8], and bidirectional encoder representations from transformers (BERT)-CRF [9]. Typically, the learning-based models perform better than the rule-based models due to the difficulty in building an “ideal” rule set.

More recently, the strategy of combining different models was widely adopted, bringing rule-based methods back to the stage. The ensemble approach can take the advantage of different models by finding the best submodel for each case. Previously proposed learning-based models as well as the rule-based models have become candidates of submodels. Taking the i2b2 shared tasks as an example, most participants presented ensemble solutions with different models involved. Among them, Liu et al [10] and Dehghan et al [11] both used rules for some categories and CRF for others in the 2014 challenge. Their rule-based taggers had better precision but inferior recall and was reported effective only for structured PHI like phone numbers. In the 2016 i2b2 shared task, ensemble with rule-based models became more popular. Lee et al [12], Dehghan et al [13], Bui et al [14], and Liu et al [15] all employed rule-based models as a component of their hybrid systems. However, despite the wide use of rules, all the works did not investigate the effect of rule-based models in hybrid architecture. Therefore, it remains an open issue if the rule-based method should be included in the ensemble approach to deidentification

### Technical Challenges

For the ensemble approach, a well-recognized opinion is that the performance of a hybrid system depends on not only the performance of submodels but also the diversity between them. Rule-based methods are usually proven inferior to popular machine learning models in terms of accuracy, which is supposed to hurt the ensemble model. Meanwhile, it was revealed that rules are substantially different from the learning-based models, which could bring a positive impact on the ensemble model. In fact, experimental results [16] provide an inconsistent observation on rule models in ensemble learning, revealing the challenge of determining the best use of the rule-based method in deidentification. It is perceivable that a weak rule-based tagger would generate noisy results and constrain the power of hybrid systems despite the diversity of rule-based models. The challenge is to determine if there is a solution to boost the ensemble approach with a proper rule-based model, which could enhance the performance with negligible cost.

### Objectives

In this paper, we present a novel ensemble approach with a rule-based component that top-performed on the 2014 i2b2 deidentification dataset, as well as an examination on the contribution of rule-based models to this task. Our system follows the idea of stacked generalization [17] and employs an ensemble classifier to combine the outputs of two learning-based subtaggers and a rule-based subtagger. We apply a transformation-based error-driven learning (TBED) algorithm [18] to automatically build a powerful rule-based model, and further explore the rule-based model’s effect on a hybrid deidentification system. Experiments show that rule-based models have a notable impact on overall performance; we can boost the F score up to 96.76% with TBED, exceeding the top performance reported in the literature so far.

## Methods

### Overview

**Figure 1:**
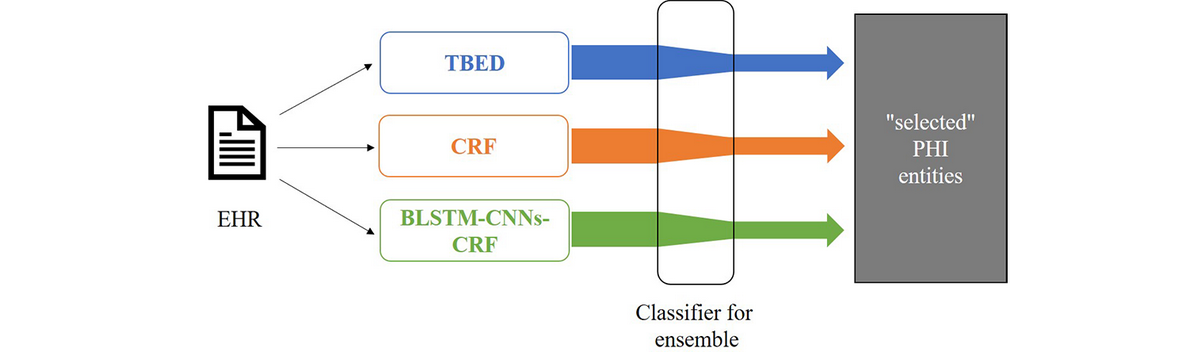
An overview of our deidentification system. BLSTM: bidirectional long short-term memory; CNN: convolutional neural network; CRF: conditional random field; EHR: electronic health record; PHI: protected health information; TBED: transformation-based error-driven learning.

In this section, we describe our system in detail. As shown in (Figure 1), the system is implemented under the framework of ensemble learning, combining two learning-based submodels and a rule-based submodel. Unlike other preliminary explorations, our discussion is centered on a data-driven algorithm that can learn the rules automatically. For a fair comparison with the existing works, we do not change the candidates of learning-based submodels, involving only CRF and LSTM-CRF. The outputs from different models are finally combined with a binary classifier that selects positive PHI entities from predicted PHI candidates.

### Rule-Based Approach

Rule-based taggers depend on precise and detailed rules; developing this type of model usually requires domain expertise. To minimize the cost to formulate such rules for deidentification, we leverage the TBED algorithm, which learns rules automatically according to their gains in correcting tagging errors. The following is the pseudocode of the TBED algorithm.


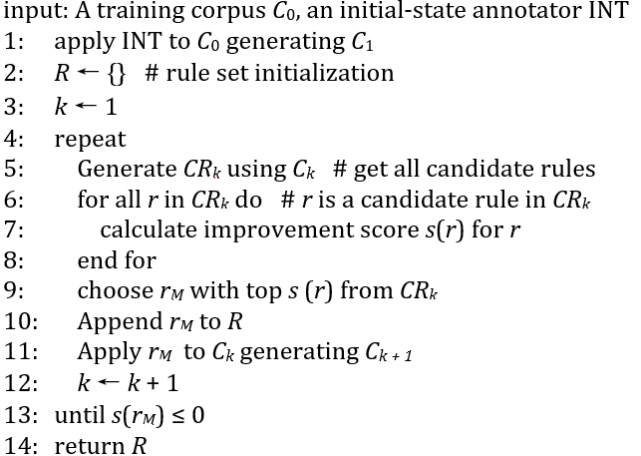


According to the TBED algorithm, at the beginning we need to define an initial annotator (INT). This annotator simply plays the role of providing a tag sequence to *S*, so it does not have to be sophisticated. In our implementation, we mine some typical regex patterns and build initial-state annotators upon them. Part of our regex patterns are shown in Table 1.

**Table 1:** Part of the patterns used in the initial-state annotator.

Regular pattern	Tag
[A-Za-z]{2,3} [0-9]{2,3}	B(USERNAME)
Hospital|HOSPITAL	I(HOSPITAL)
\w+@\w+\.[A-Za-z]{3}	B(EMAIL)
St|Street|Avenue|Lane|Drive|Rd|Road|Circle|Place	I(STREET)
\d{4}|\d{2}-\d{2}-\d{2}|\d{4}	B(DATE)

After applying the initial tagger, the main body of TBED (from line 4 of the TBED algorithm) starts to collect the most profitable transformation in all possible transformations. In line 5, if a tag *t_i_* doesn’t match the correct tag *t_g_* at the *i*th position, a candidate rule changing *t_i_* to *t_g_* is generated (eg, *if current token is s_i_* and *if the length of previous token is l_i_*_– 1_*, then change t_i_ to t_g_*). The transformations can be conditional on different features (see also the section Unified Feature Set) from different perspectives, forming a group of candidate rules (*CR_k_*). From line 6 to line 8, we scanned each rule through the corpus to determine its benefit *s(r)* according to the tags in *C_k_*. Then from line 9 to line 11, the rule with the best score is chosen to be used in the generated tagger and is appended to an ordered list of rules at each iteration. This rule set can be further improved by another round of iteration. After leveraging this greedy searching strategy several times, we can get many helpful transformation rules, resulting in a greatly empowered rule-based tagger.

### Learning-Based Models

The learning-based models are dominating in the recent deidentification research. Among them, two models always appear in the center stage: one is CRF, the other is neural network. Accordingly, we built two different types of models based on CRF and RNN, respectively, and integrated them into the hybrid system.

The CRF models *P* (*T* │ *S*) using a Markov random field, with nodes corresponding to elements of *T*, and the potential functions are conditional on (features of) *S*. CRF offers several advantages over the hidden Markov model (HMM), including the ability to relax strong independence assumption made in the HMM. Moreover, CRF also avoids a fundamental limitation of maximum entropy Markov models (MEMMs), which can be biased towards states with few successor states. One common use of CRF is sequence labeling problems like named entity recognition (NER), in which case the Markov field is a chain and the CRF predicts the most possible *T* conditioned on the input sequence *S* via equation 1.


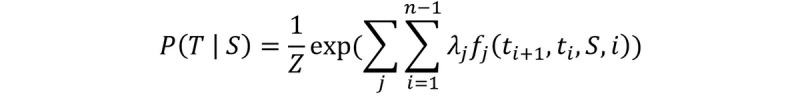


(1)

In equation 1, *f_j_*(*t_i_*_+ 1_, *t_i_*, *S*, *i*) is a feature function, 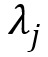 is a learnable weight for the feature function, and *Z* is the normalization factor. Feature functions are usually defined as indicator functions. For example, a feature function may have a value of 0 in most cases, and a value of 1 if a feature of *t_i_*_+ 1_ is 1 (eg, the length of *t_i_*_+ 1_ is 4) and a feature of *t_i_* is 2 (eg, *t_i_* is a punctuation). 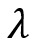 can assign the weight of such a feature function.

The neural network (NN)-based one is similar to the BLSTM-CNNs-CRF architecture proposed by Ma et al [19]. It first builds a dense representation of the input sequence by concatenating word embeddings with character embeddings extracted by a convolutional neural network (CNN) layer. This representation is then fed into a bidirectional LSTM encoder, and a CRF layer is employed as the last layer to predict the most probable tag. We modified this model by adding feature embedding to the input, providing more information to the downstream LSTM-CRF network. We omit the details of this model and refer readers to Ma et al [19] for brevity.

### Unified Feature Set

As features for the submodels, a unified feature set was constructed. According to previous explorations and our experiments on this data, we chose the following 3 types of features.

- Token-level features: length of the token; whether the token contains only numbers; whether the token starts with an uppercase letter; the stem, prefix, suffix of the token; etc
- Global features: sentence length, section information [15]
- Tagging-based features: general NER tag and part of speech (POS) tag from Stanford CoreNLP [20]

### Ensemble Method

Ensemble learning is a technique that combines multiple models to obtain better predictive performance. In the 2014 i2b2 deidentification challenge, 4 of 8 participants used the ensemble of rules and CRFs, and the overall top 3 systems were hybrid systems. For deidentification, ensemble is always performed at the output layer (ie, combining the outputs from the submodels). The most popular and successful ensemble strategy in the challenge is using rules for some categories and CRFs for others. Although it proved useful in the challenge, there are still many shortcomings for this method. The division of categories are manually made based mainly on intuition, and the category-level choice is inflexible, which misses details of different samples. To avoid these shortcomings, we chose a fine-grained learning-based ensemble method: stacking.

Following Kim et al [21], we combined the predictions of the rule-based model and learning-based models via stacked generalization. Specifically, the predicted PHI from submodels are fed into a binary SVM-based classifier to make the decision about which PHI is more likely to be correct. The ensemble learner scores PHI according to some features (eg, which predictor(s) predicted this PHI, the overlap with other PHI, the type of this PHI) and picks PHI with higher scores.

## Results

### Data Sets and Evaluation Metrics

In the 2014 i2b2 deidentification shared task, a corpus of clinical narratives were released with PHI expressions, consisting of 1304 English medical records for 296 patients with 805,118 whitespace-separated tokens [22]. The 2014 i2b2 deidentification data set was manually annotated with a total of 28,867 PHIs. The PHI categories defined by HIPAA are extended into 23 fine-grained PHI subcategories (the i2b2 category hereafter). Detailed PHI distributions are shown in Table 2. Note that the corpus is divided into a training set and a testing set, with 790 and 514 records, respectively.

**Table 2:** Protected health information (PHI) distribution in the 2014 i2b2 deidentification corpus (total PHI in training set=17,405 and total PHI in test set=11,462).

HIPAA^a^ categories and i2b2^b^ categories		Training set	Test set
**DATE**			
	DATE	7502	4980
**NAME**			
	DOCTOR	2885	1912
	PATIENT	1316	879
	USERNAME	264	92
**AGE**			
	AGE	1233	764
**CONTACT**			
	PHONE	309	215
	FAX	8	2
	EMAIL	4	1
	URL	2	0
**ID**			
	MEDICALRECORD	611	422
	IDNUM	261	195
	DEVICE	7	8
	BIOID	1	0
	HEALTHPLAN	1	0
**LOCATION**			
	HOSPITAL	1437	875
	CITY	394	260
	STATE	314	190
	STREET	216	136
	ZIP	212	140
	ORGANIZATION	124	82
	COUNTRY	66	117
	LOCATION-OTHER	4	13
**PROFESSION**			
	PROFESSION	234	179

^a^HIPAA: Health Insurance Portability and Accountability Act.

^b^i2b2: Informatics for Integrating Biology and the Bedside.

Evaluation metrics are selected as the popular precision (P), recall (R) and F1-measure (F1) as illustrated by equation 2. The primary metric of this shared task is the entity-level strictly matched F1 score, which requires that the start, end, and class under i2b2 categories are all matched with the golden annotation. The organizers provided an evaluation script to calculate this score [23]. To make our experiments comparable with baselines, all the results are evaluated using this script.


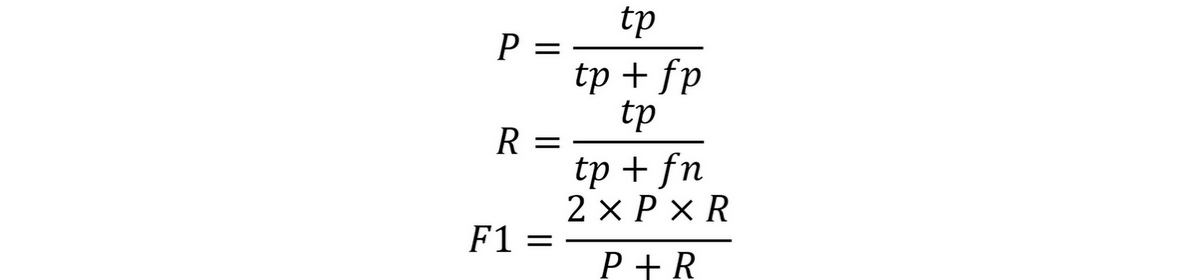


(2)

### Preprocessing and Experimental Setups

The whitespace-separated tokens do not exactly match the PHI in the i2b2 corpus (ie, there is PHI starting or ending in the middle of a token), making them impossible to be correctly annotated under the token-level IOB scheme. For example, token “*Dr.Smith*” contains the PHI “*Smith*”, but a token-level tagger can only annotate the entire string “*Dr.Smith*” as an entity and never outputs the correct PHI “*Smith*”, which hurts performance severely. This is the reason why subword level tokenization is necessary. We performed the following steps for tokenization to tackle this problem. First, all characters are split except continuous letters and continuous numbers, which are less likely to be the start or end of a PHI. Second, the continuous letters are further split at the position of uppercase letters. Third, we run byte pair encoding (BPE) on the tokenized corpus to alleviate data sparseness. For example, the string “48-year-old in Edwin HealthCare” will be tokenized as (48, -, year, -, old, in, Edwin, Health, Care). This reduced the error rate of tokenization regarding PHI to 0.22%.

We performed 10-fold cross-validation to tune the hyper-parameters. TBED outputs 43 transformation rules from 43 iterations. CRF uses an extended feature set with 49 different types of feature crosses. We used linear_chain_crf [24] as the implementation of CRF, which can use a graphics processing unit (GPU) to accelerate. The BLSTM-CNNs-CRF model is implemented with TensorFlow [25]. The SVM-based ensemble learner uses radial basis function (RBF) kernel with LIBSVM [26]. Other hyper-parameters are shown in Table 3.

**Table 3:** The hyper-parameters setting.

Hyper-parameter	Value
Learning rate for conditional random field	0.0005
Regularization weight	0.0003
Kernel size for CNN^a^	2, 3, 4, 5
Number of channels of CNN	8
Dimension of character embedding	16
Dimension of word embedding	128
Dimension of feature embedding	4 per feature
LSTM^b^ hidden size	128
Gradient clip	10
Learning rate for LSTM	0.0002
SVM^c^ C value for positive samples	5.2
SVM C value for negative samples	12.48
SVM gamma value	0.009

^a^CNN: convolutional neural network.

^b^LSTM: long short-term memory.

^c^SVM: support vector machine.

### Statistical Results

In this section, we report the results of our experiments. The results of our models as well as a comparison with baselines are shown in Table 4. We selected three representative previous works as our baselines. Yang et al [27] is the winner of the 2014 i2b2 deidentification challenge, they employed rules for some types of PHI and CRFs for others. Liu et al [15] is a representative work on ensemble learning, which consists of 3 learning-based models, CRF, LSTM-CRF, and LSTM-CRF-FEA (feature), where the LSTM-CRF-FEA takes hand-crafted features as additional inputs. The main difference between Liu et al [15] and our study is that they did not combine a rule-based model. Besides, they used a smaller feature set with no feature crosses for the CRF. Beryozkin et al [28] is the state-of-the-art (SOTA) solution on the 2014 i2b2 data set. They used a BiRNN-CRF model with character-level RNNs and achieved an F1 of 96.00%.

**Table 4:** Results of the hybrid system and submodels (i2b2 categories, strict entity matching).

Model	Precision, %	Recall, %	F1-measure, %
Yang et al [27] (CRF^a^ + Rule)	96.45	90.92	93.60
Liu et al [15] (CRF + LSTM^b^*2)	96.46	93.80	95.11
Beryozkin et al [28] (BiRNN^c^)	—^d^	—^d^	96.00
Rule-based	91.92	90.36	91.13
CRF	97.58	93.30	95.39
BLSTM^e^-CNNs^f^-CRF	96.91	95.74	96.32
Ensemble	98.15	95.41	96.76

^a^CRF: conditional random field.

^b^LSTM: long short-term memory.

^c^RNN: recurrent neural network.

^d^These results are not reported in the original paper.

^e^BLSTM: bidirectional long short-term memory.

^f^CNN: convolutional neural networks.

As for our models, the rule-based submodel achieved a satisfactory F1 score of 91.13%; the CRF-based submodel is more powerful with an F1 score of 95.39%; and the NN-based submodel is about 1% better than the CRF-based model with an F1 score of 96.32%. The final result of our ensemble system was 96.76%, achieving a new SOTA system.

To discuss whether TBED is a good solution to rule-based deidentification, a comparison of our data-driven rule-based model and other hand-crafted rule-based models is shown in Table 5. Two distinguished rule-based methods in the 2014 i2b2 competition are selected. The first is Liu et al [10] using regular expressions to identify standardized PHI such as PHONE, FAX, and EMAIL with one pattern per category. Their system achieved a high precision of 97.92% but a low recall of 1.64%, making the averaged F1 only 3.23%. The second is Dehghan et al [11] leveraging dictionaries and more sophisticated rules. With undisclosed manual cost, they achieved an 87.53% F1 score for part of the PHI categories, which is the best-performed rule-based results reported in the literature. We applied TBED to all 23 PHI categories and achieved an F1 score of 91.13%.

**Table 5:** Results of rule-based taggers (i2b2 categories, strict entity matching).

Method	Precision, %	Recall, %	F1-measure, %
Liu et al [10] (Regex)	97.92	1.64	3.23
Dehghan et al [11] (dictionary + rules)^a^	89.68	85.91	87.53
Our method, initial-state tagger (Regex)	69.28	33.53	45.19
Our method (Regex + TBED^b^)	91.92	90.36	91.13

^a^Only part of the personal health information categories were counted, resulting in a higher recall.

^b^TBED: transformation-based error-driven learning.

We also explored the components in our TBED method. There are two parts in our rule-based model: the initial-state tagger (based on Regex) and the transformation-based tagger (TBED). As shown in Table 5, although our initial-state tagger performs poorly with an F1 of 45.19%, it could be rapidly improved to 91.13% after 43 rounds of iteration.

To further verify the impact of each submodel, especially the role of TBED in the ensemble learning, we performed an ablation study by removing each component of the hybrid system. The corresponding performances are shown in Table 6. If we exclude BLSTM-CNNs-CRF from the hybrid system, the F1 becomes 96.07% with a decrease of 0.69%. When we remove the rule-based model, the ensemble of learning-based models can only reach an F1 of 96.42%, and it can be improved back to 96.46% by recovering the initial-state tagger. CRF has the least impact of 0.1% from 96.76% to 96.66%.

**Table 6:** Results of the hybrid system without submodels (i2b2 categories, strict entity matching).

Model	F1-measure, %	Change, %
Ensemble	96.76	0
Without TBED^a^ (with Regex)	96.46	–0.30
Without TBED (without Regex)	96.42	–0.34
Without CRF^b^	96.66	–0.10
Without BLSTM^c^-CNNs^d^-CRF	96.07	–0.69

^a^TBED: transformation-based error-driven learning.

^b^CRF: conditional random field.

^c^BLSTM: bidirectional long short-term memory.

^d^CNN: convolutional neural network.

## Discussion

### Analysis of Principal Results

The results of our system were quite positive. Our rule-based model achieved an F1 of 91.13%, which surpasses the existing practices in rule-based deidentification. From the comparison of Regex and Regex with TBED, we found that TBED is not necessarily dependent on a fine-tuned initial tagger. In other words, TBED could efficiently learn a rule-set to best approximate the training data. The performance of our CRF model was an F1 of 95.39%, which outperforms the previous hybrid systems. We believe that this improvement is mainly from the more detailed feature set and feature crosses between the features. The BLSTM-CNNs-CRF also showed advantage over the BiRNN model presented by Beryozkin et al [28] with a gap of 0.32% in F1, which is the best performing submodel. Integrating them together, our ensemble framework improved the best performing submodel BLSTM-CNNs-CRF by about 0.4% in F1. The improvement of a hybrid system is usually from the diversity of its components. Table 7 shows some cases of the difference between submodels, which may reveal where the improvement comes from. Opposite to the learning-based models, which are optimized to generalize the whole data set, rule-based models usually focus on a specific condition, which offers the ability to deal with rare cases.

**Table 7:** Examples of transformation-based error-driven learning contribution to ensemble result.

Cases	TBED^a^	CRF^b^	BLSTM^c^-CNNs^d^-CRF	Ensemble	Golden standard
with SVR^e^ of *1739*^f^	—^g^	DATE	DATE	—	—
family contact: *Talissa Irish*	PATIENT	—	—	PATIENT	PATIENT
Patient Name: FOUST,FAY [*50294530*(LHCC)]	RECORD	—	PHONE	RECORD	RECORD
a CK^g^ of *1028*	—	DATE	DATE	—	—
go back to *New* *Jersey*	STATE	—	HOSPITAL	STATE	STATE
739 Newburgh Street, Sulphur, AR *26822*	ZIP	—	RECORD	ZIP	ZIP

^a^TBED: transformation-based error-driven learning.

^b^CRF: conditional random field.

^c^BLSTM: bidirectional long short-term memory.

^d^CNN: convolutional neural network.

^e^SVR: systemic vascular resistance

^f^Italics indicate the protected health information for each case.

^g^Not a privacy entity.

^h^CK: creatine kinase

The results of our ensemble system also showed advantages over all previous explorations. Compared with previous top performing hybrid systems (Yang et al [27] and Liu et al [15]), our system offers significant improvements of ≥1.5% in all the metrics. It also creates a new SOTA system that exceeds the previous SOTA of 0.76%, further proving the effectiveness of our approach.

### Interpretations of Ablation Study

From the results shown in Table 6, we can observe that removing any submodel will hurt performance, indicating that the three submodels contribute to the task rather than bring the redundancy. It is natural to observe that the top performing BLSTM-CNNs-CRF submodel has the greatest impact on ensemble results. An amazing discovery is that TBED ranks as second in influence on overall performance, despite it being the least performed single model. This confirms that a rule-based tagger is more indispensable to the hybrid system than another learning-based submodel. We further examined the components in TBED; it was enlightening to find that the initial tagger (Regex) itself was still beneficial to the final results. This consolidate that even a small part of high-quality rules can be informative to the ensemble model.

To sum up, we found that the performance of rule-based models does not affect overall results, and even an advanced hybrid system with few upside potentials can be further improved by a rule-based model. Although the rule-based model with TBED seems to be a weaker tagger compared with learning-based models, it can still provide information useful for the ensemble model.

### Conclusions

In this paper, we introduced a new hybrid system for the anonymization of EHRs, boosted by a rule-based tagger that can automatically search transformation rules via TBED. The ensemble system contains three submodels based on rules, CRF, and NN, and is integrated by SVM-based stacking. In the experiments, we found that a hybrid deidentification system can be boosted by a rule-based model with TBED, achieving top performing results for this task. We also performed an ablation study to prove the necessity of the rule-based submodel with TBED steps, which further proves the accuracy of our findings.

In the future, we will explore the more detailed difference between rule-based models and learning-based models. Possible directions are checking their performance on various categories and analyzing the interactions between different models. We will also take more models into account and check the effect of rules on more powerful models such as the recent astonishing pretrained models like BERT [29].

## Abbreviations

BERT: bidirectional encoder representations from transformers
BPE: byte pair encoding
CNN: convolutional neural network
CRF: conditional random field
EHR: electronic health record
FEA: feature
F1: F1-measure
GPU: graphics processing unit
HIPAA: Health Insurance Portability and Accountability Act
HMM: hidden Markov model
i2b2: Informatics for Integrating Biology and the Bedside
INT: initial annotator
IOB: inside, outside, and begin
LSTM: long short-term memory
MEMM: maximum entropy Markov model
NER: named entity recognition
NN: neural network
P: precision
PHI: protected health information
POS: part of speech
R: recall
RBF: radial basis function
RNN: recurrent neural network
SOTA: state-of-the-art
SVM: support vector machine
TBED: transformation-based error-driven learning
